# Serum Calprotectin Level Is Independently Associated With Carotid Plaque Presence in Patients With Psoriatic Arthritis

**DOI:** 10.3389/fmed.2022.932696

**Published:** 2022-07-08

**Authors:** Isaac T. Cheng, Huan Meng, Martin Li, Edmund K. Li, Priscilla C. Wong, Jack Lee, Bryan P. Yan, Alex P. W. Lee, Ho So, Lai-Shan Tam

**Affiliations:** ^1^Department of Medicine and Therapeutics, Prince of Wales Hospital, The Chinese University of Hong Kong, Shatin, Hong Kong SAR, China; ^2^Jockey Club School of Public Health and Primary Care, The Chinese University of Hong Kong, Hong Kong, Hong Kong SAR, China

**Keywords:** psoriatic arthritis, serum calprotectin, subclinical atherosclerosis, carotid ultrasound, LASSO regression

## Abstract

**Background:**

Whether calprotectin could play a role in augmenting cardiovascular (CV) risk in patients with psoriatic arthritis (PsA) remains uncertain. The aim of this study is to elucidate the association between serum calprotectin level and subclinical atherosclerosis in patient with PsA.

**Method:**

Seventy-eight PsA patients (age: 52 ± 10 years, 41 [52.6%] male) without CV disease were recruited into this cross-sectional study. Carotid intima-media thickness (cIMT) and the presence of plaque were determined by high-resolution ultrasound. Calprotectin levels in serum were quantified by enzyme-linked immunosorbent assay. The variables associated with the presence of carotid plaque (CP) were selected from the least absolute shrinkage and selection operator (LASSO) regression analysis.

**Results:**

29/78 (37.2%) of patient had carotid plaque (CP+ group). Serum calprotectin level was significantly higher in the CP+ group (CP− group: 564.6 [329.3–910.5] ng/ml; CP+ group: 721.3 [329.3–910.5] ng/ml, *P* = 0.005). Serum calprotectin level correlated with PsA disease duration (rho = 0.280, *P* = 0.013) and mean cIMT (rho = 0.249, *P* = 0.038). Using LASSO regression analysis, the levels of Ln-calprotectin (OR: 3.38, 95% CI [1.37, 9.47]; *P* = 0.026) and PsA disease duration (OR: 1.09, 95% CI [1.01, 1.18]; *P* = 0.013) were screened out from a total of 19 variables. The model in predicting the presence of CP was constructed by Ln-calprotectin and PsA disease duration with an area under the receiver-operating characteristic (ROC) curve of 0.744, (95 CI% [0.59, 0.80], *P* = 0.037).

**Conclusion:**

Serum calprotectin level is associated with the presence of CP in PsA. Further studies are required to confirm whether this pathway is associated with CV events in PsA.

## Introduction

Psoriatic arthritis (PsA) is a systemic inflammatory arthropathy associated with an increased cardiovascular disease (CVD) risk (1, 2). The prevalence of traditional CV risk factors is higher in PsA patients when compared to the general population (1, 3, 4). Nonetheless, traditional CV risk factors can only explain part of the elevated risk for myocardial infarction in PsA patients (1). Different types of inflammatory arthritis had no significant difference in the incidence and prevalence of major adverse cardiovascular events, indicating that inflammation *per se* may contribute to the CV risk rather than a specific disease (5). Nonetheless, data exploring the mechanisms of how inflammation increase CV risk in PsA patients are limited.

We have demonstrated that the prevalence of subclinical coronary and carotid atherosclerosis was also increased in PsA (6, 7). The presence of carotid plaque (CP) independently predicted the risk of CV events after adjusting for traditional CV risk scores and inflammatory burden (as reflected by the Disease Activity in Psoriatic Arthritis [DAPSA]), with hazard ratio (HR) ranging from 2.35 to 3.42 (8). As all the CV risk scores underestimated the subclinical carotid atherosclerosis risk, and the European League Against Rheumatism (EULAR)-recommended modification only improved the sensitivity of the CV risk scores to a moderate level (9), soluble biomarkers which are associated with carotid atherosclerosis may be useful to guide physicians to whom carotid ultrasound should be considered. Biomarkers such as soluble ST2, which acts as a decoy receptor for interleukin-33, were independently associated with the presence of CP in PsA (10). Yet, soluble ST2 level did not correlate with any inflammatory markers (10). Biomarkers that are associated with metabolic abnormalities and dysregulated inflammatory pathways which drive the atherosclerotic process in PsA deserve further studies.

Calprotectin (S100A8 and S100A9), the most-investigated S100 protein in rheumatic diseases by far, is recognized by toll-like receptor four on multiple inflammatory cell types which promote vascular plaque formation and destabilization through upregulating inflammatory cytokine expression (11–13). Serum calprotectin level was an early and sensitive marker of unstable angina (14), and was associated with CV events (15). Whether calprotectin might play a role in accelerating atherosclerotic disease secondary to systemic inflammation is worth exploring.

Rheumatoid arthritis and PsA patients had higher serum calprotectin level (16, 17). In patients with inflammatory arthritis treated with anti-tumor necrosis factor-α (anti-TNF-α), calprotectin levels were associated with the progression of aortic pulse wave velocity (baPWV) (18). Patients with CP occurrence were also reported a higher serum calprotectin level (19). Whether serum calprotectin level correlates with inflammatory burden and subclinical atherosclerosis in PsA would need to be addressed.

The objective of our study was to determine the relationship between serum calprotectin level, subclinical atherosclerosis and inflammatory burden in PsA patients.

## Materials and Methods

### Study Design and Patients

Psoriatic arthritis patients who fulfilled the Classification of Psoriatic Arthritis criteria were recruited consecutively for this cross-sectional study from the rheumatology clinic of The Prince of Wales Hospital (PWH). Exclusion criteria were pregnancy and patients who had prior CV events. This study was approved by the Ethics Committee of The Chinese University of Hong Kong and conducted according to the ICH-GCP guidelines. All study participants signed an informed consent in accordance with the Declaration of Helsinki.

### Clinical Interview and Laboratory Tests

Psoriatic arthritis disease features were recorded including pain, physician’s and patient’s global assessment, 68 tender joint count, 66 swollen joint count, the irreversibly 68 damaged joint count, the dactylitis counts, and the Maastricht Ankylosing Spondylitis Enthesitis Score (MASES, range from 0 to 13) and Health Assessment Questionnaire Disability Index (HAQ-DI). Blood levels of C-reactive protein (CRP) and erythrocyte sedimentation rate (ESR) were documented. Minimal disease activity was chosen as the treatment target (20). Joint and skin disease activity were assessed using the Psoriasis Activity and Severity Index (PASI) and DASPA, respectively.

### Cardiovascular Assessments

The following anthropometric assessments were performed for all patients before the carotid ultrasound (US) exam: body height and weight, body mass index (BMI), waist and hip circumferences. Current smoking and alcohol status, past clinical and medication use history, and family history were retrieved from the medical record. Fasting blood glucose and lipid profile were also taken. Hypertension was defined as systolic blood pressure ≥ 140 mmHg or diastolic blood pressure ≥ 90 mmHg with two consecutive records in the hospital, and/or current antihypertensive drugs use. A diagnosis of hyperlipidemia was considered if the total cholesterol was ≥ 6.2 mg/dL or LDL-cholesterol was ≥ 4.13 mg/dL or current statin use. Framingham risk score (FRS) was used for assessing the 10-year CVD risk and was classified into three categories: low-risk < 10%, intermediate-risk 10–19%, and high-risk ≥ 20%.

### Subclinical Atherosclerosis Assessment

All participants had the carotid intima-media thickness (cIMT) and plaque assessment done at the PWH. Measurements of cIMT were carried out using a high-resolution B-mode US machine (Philips EPIQ7) as described previously (10) and were performed by an experienced sonographer blinded to all patients’ information. Bilateral distal common carotid artery, bulb, and proximal internal carotid artery were assessed. The presence of plaque was detected and the mean/maximum cIMT of 6 arterial segments was computed accordingly. The reproducibility of cIMT in our center was 0.97 (19). As described before, CP criteria were defined as the focal protrusion of cIMT > 1.2 mm that did not involve the whole lumen uniformly (20).

### Serum Calprotectin Measurement

Serum calprotectin level was analyzed by the QUANTA Lite Calprotectin Extended Range ELISA kit (INOVA Diagnostics, San Diego, CA, United States). All the detection procedures followed the manufacturer’s protocol. All samples from each patient were studied in parallel on the same plate at the same time. The normal range of calprotectin was 0.1 to 1.6 μg/ml (21, 22).

### Statistical Analysis

Baseline anthropomorphic assessments were described as mean ± SD, median (interquartile range), or percentage (%) according to the presence of CP (CP+ vs. CP−). Comparisons and correlation analysis were performed using the independent samples *t*-test or Mann-Whitney *U* test and Pearson’s or Spearman’s correlation for continuous variables, and Chi-squared or Fisher’s exact test for categorical variables according to the distribution of data.

The least absolute shrinkage and selection operator (LASSO) regression analysis, a shrinkage method of variable selection for linear regression models was also performed to develop the predictive model for presence of carotid plaque. The variables with non-zero coefficients after the penalized procedures were further studied. To centralize and normalize the variables which were put into the LASSO regression analysis, the 10-fold cross-validation was used as the resampling method. Lambda.1se was chosen as the best lambda for its ability to choose the most concise variables with a good performance of predictive model. The remaining variables were used to construct the predictive model which was presented by a nomogram diagram validated with a 100 times bootstrap resampling method (23). The receiver characteristic curve (ROC) curve was used to assess the utility of calprotectin in discriminating patients with and without CP. The calibration curve and the Hosmer-Lemeshow test was used to test the goodness of fit of the model. The dplyr, glmnet, pROC, rms, ResourceSelection package of R studio^1^ (version 3.6.1) were used to perform the analyses and ggplot package was used to create figures. Two-sided *P* values < 0.05 were considered to indicate statistical significance.

## Results

### Demographic Characteristics of Psoriatic Arthritis Patients

A total of 78 (41 male, 52.6%) PsA patients were recruited. The mean age was 52 ± 10 years, and the mean PsA disease duration was 14.1 ± 7.0 years. The cohort of patient had mild disease activity (mean DAPSA 4.7 ± 5.1) and moderate CV risk (FRS 10.7 ± 8.4). The mean serum calprotectin level was 778.3 ± 567.8 ng/ml (Table 1).

**TABLE 1:** Anthropometric and clinical characteristics of PsA patients with or without carotid plaque.

		Carotid plaque				
	
	All (*N* = 78)	No (*n* = 49)	Yes (*n* = 29)	*P*-value
Male gender, n (%)	41 (52.6%)	22 (44.9%)	19 (65.5%)	0.078
Age, (years)	52 ± 10	50 ± 11	56 ± 8	0.024*
**PsA characteristics**				
PsA disease duration, (years)	14.1 ± 7.0	12.4 ± 7.0	17.0 ± 6.3	0.005*
Tender joint count, (0–68)	0.5 (0, 3)	1 (0, 3)	0 (0, 3)	0.825
Swollen joint count, (0–66)	0 (0, 2)	0 (0, 1)	1 (0, 2)	0.044*
Damaged joint count, (0–68)	2 (0, 6)	0 (0, 5)	1 (0, 8)	0.437
Dactylitis (0–20)	0 (0, 1)	0 (0, 0)	0 (0, 0)	0.091
MASES enthesitis, (0–13)	0 (0, 11)	0 (0, 1)	0 (0, 0)	0.467
VAS Pain, (0–100)	30 (14, 50)	30 (20, 50)	30 (10, 60)	0.654
PtGA, (0–100)	40 (20, 60)	35 (20, 55)	50 (30, 60)	0.129
PGA, (0–100)	20 (6, 40)	15 (6, 35)	20 (10, 40)	0.427
PASI, (0–72)	2 (0.5, 5.9)	2 (0.7, 5.8)	1.8 (0.5, 5.4)	0.922
HAQ, (0–3)	0.5 (0.0, 0.6)	0.1 (0.0, 0.4)	0.4 (0.0, 0.9)	0.149
MDA, n (%)	13 (16.7%)	10 (20.4%)	3 (10.3%)	0.249
DAPSA, (0–64)	2.5 (1.0, 6.0)	2.2 (0.9, 6.8)	2.8 (1.2, 5.1)	0.722
ESR, (mm/1st hr)	16 (7.0, 30.5)	14.5 (7.0, 29.0)	21.0 (7.0, 32.0)	0.991
CRP, (mg/l)	3.0 (1.0, 6.0)	2.8 (1.0, 5.3)	3.3 (1.1, 8.3)	0.506
**CV traditional risk factors**				
Body weight, (kg)	67.2 ± 13.1	67.9 ± 13.7	66.1 ± 12.4	0.562
BMI, (kg/m^2^)	25.2 ± 4.1	25.6 ± 4.2	24.6 ± 3.9	0.314
Waist-to-hip ratio	0.9 ± 0.1	0.9 ± 0.1	0.9 ± 0.1	0.544
Current smoker, n (%)	8 (10.2%)	5 (10.2%)	3 (10.3%)	0.248
Current drinker, n (%)	24 (30.8%)	16 (32.6%)	8 (27.6%)	0.622
Systolic BP, (mmHg)	127 ± 15	126 ± 15	128 ± 15	0.765
Diastolic BP, (mmHg)	82 ± 11	81 ± 10	83 ± 12	0.483
Total cholesterol, (mmol/l)	5.0 ± 0.8	4.9 ± 0.8	5.1 ± 0.8	0.350
HDL cholesterol, (mmol/l)	1.4 ± 0.4	1.4 ± 0.3	1.5 ± 0.5	0.481
LDL cholesterol, (mmol/l)	3.0 ± 0.7	2.9 ± 0.7	3.1 ± 0.7	0.206
Triglycerides, (mmol/l)	1.4 ± 0.9	1.5 ± 1.0	1.3 ± 0.5	0.196
Fasting plasma glucose, (mmol/l)	5.5 ± 1.5	5.7 ± 1.8	5.1 ± 0.7	0.155
Hypertension, n (%)	42 (53.8%)	27 (55.1%)	15 (51.7%)	0.772
Hyperlipidemia, n (%)	19 (24.3%)	9 (18.4%)	10 (34.5%)	0.109
Diabetes, n (%)	14 (17.9%)	9 (18.4%)	5 (17.2%)	0.900
FRS, (%)	10.7 ± 8.4	9.2 ± 8.5	12.7 ± 7.8	0.094
**Current medications, n (%)**				
Anti-hypertensive	39 (50.0%)	26 (53.1%)	13 (44.8%)	0.482
Statins	12 (15.4%)	5 (10.2%)	7 (24.1%)	0.099
NASIDs	35 (44.9%)	23 (36.9%)	12 (41.4%)	0.633
csDMARDs	45 (57.7%)	30 (61.2%)	15 (51.7%)	0.412
bDMARDs	13 (16.7%)	8 (16.3%)	5 (17.2%)	0.917
**Biomarker**				
Serum calprotectin, (ng/ml)	665.4 (415.0, 947.0)	564.6 (329.3, 910.5)	721.3 (574.1, 1268.4)	0.005*

*P value < 0.05. VAS, visual analog scale; PtGA, patients’ global assessment of disease activity; PGA, physicians’ global assessment of disease activity; PASI, psoriasis area and severity index; HAQ, health assessment questionnaire; DAPSA, disease activity in psoriatic arthritis; MDA, minimal disease activity; CRP, C-reactive protein; ESR, erythrocyte sedimentation rate; BMI, body mass index; BP, blood pressure; FRS, Framingham risk score; HDL, high-density lipoprotein; LDL, low-density lipoprotein; NSAID, non-steroidal anti-inflammatory drugs; csDMARDs, conventional synthetic disease-modifying antirheumatic drugs. bDMARDs, biologics disease-modifying antirheumatic drugs. Values are the number (percentage) or median (interquartile range) or mean ± SD.

### Serum Calprotectin Level and Inflammatory Burden

There was a positive correlation of serum calprotectin level with PsA disease duration (rho = 0.280, *P* = 0.013). The relationship between serum calprotectin level and CRP levels (rho = 0.176, *P* = 0.127), ESR levels (rho = 0.044, *P* = 0.704). and DASPA (rho = 0.135, *P* = 0.243) was not significant (Supplementary Table 1).

### Serum Calprotectin and Carotid Atherosclerosis

**FIGURE 1:**
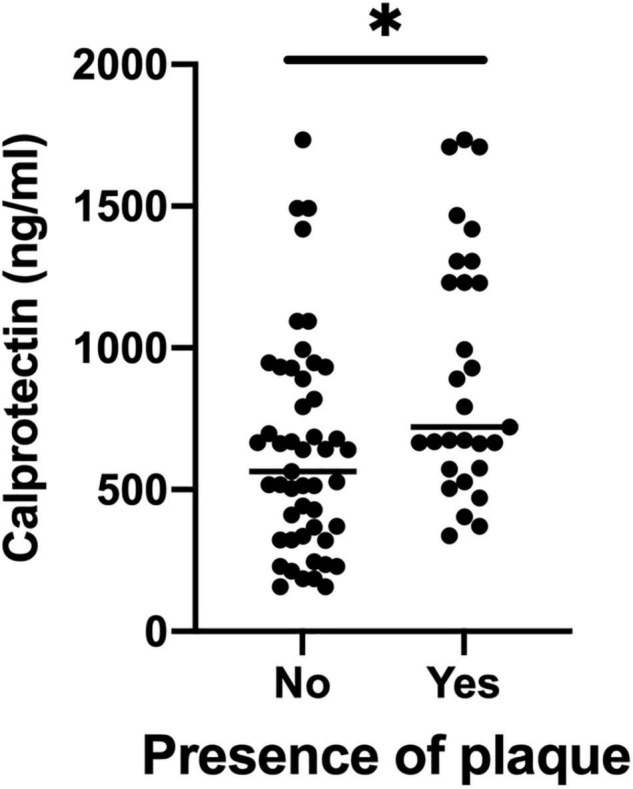
Serum calprotectin levels between CP+ groups and CP– groups. CP, carotid plaque. *P value < 0.05.

The mean cIMT in this cohort was 0.62 ± 0.10 mm. Serum calprotectin was significantly correlated with mean cIMT (rho = 0.249, *P* = 0.038) (Supplementary Table 1). Twenty-nine patients (37.2%) had CP (CP+ group). The CP+ group was older, had longer disease duration and more swollen joints (Table 1), yet the traditional CV risk profile was similar compared with the CP− group (FRS in CP+: 12.7 ± 7.8 vs. CP−: 9.2 ± 8.5; *P* = 0.094). Serum calprotectin level was significantly higher in the CP+ group (721.3 ng/ml [574.1, 1268.4] vs. 564.6 ng/ml [329.3, 910.5], *P* = 0.005) (Figure 1 and Table 1).

### Predictive Model for the Presence of Carotid Plaque

**FIGURE 2:**
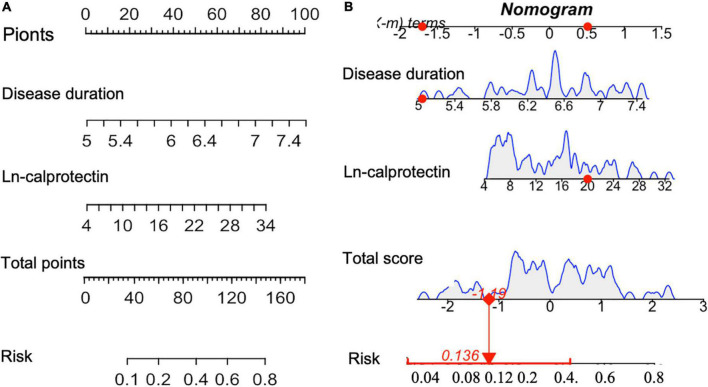
**(A)** Nomogram for predicting the probability of CP+ including the risk factors of PsA disease duration and Ln-calprotectin. To estimate the risk for the CP+ (shown as “Risk” in the figure) among patients with PsA, each individual patient’s values were plotted on each variable axis. A verticle line was drawn from that value to the top “Points” scale to determine the number of points that were assigned by that variable value. Then, the points from each variable value were summed. The sum on the “Points” scale was located and vertically projected onto the bottom “Total points” axis, and then a personalized risk of CP for PsA was obtained. CP+, the presence of carotid plaque **(B)**. The dynamic nomogram was used as an example (one patient in our sample).

Least absolute shrinkage and selection operator regression was performed to screen out predictive variables including age, gender, traditional CV risk factor, clinical features, disease activity and medication use (total 19 variables) as shown in Supplementary Table 2. Two (Ln-calprotectin and PsA disease duration) of 19 variables were selected (Lambda.1se = −1.99) and then used for building a predictive model (Supplementary Figure 1). Ln-calprotectin (OR: 3.38, 95% CI [1.37, 9.47]; *P* = 0.026) and PsA disease duration (1.09, 95% CI [1.01, 1.18]; *P* = 0.013) were statistically significant in predicting CP+ (Table 2). The predictive model based on the risk factors identified by the multivariate logistic regression analysis was presented as a nomogram for easy computation of the probability of having CP+ (Figure 2A). As shown in Figure 2B, a PsA patient with a disease duration of 5 years and a level Ln-calprotectin of 20 has an estimated probability of CP+ of 13.6% which was calculated directly by R studio (Figure 2B).

**TABLE 2:** The logistic regression analysis of variables selected by LASSO regression for predicting the presence of carotid plaque.

			Predictive model	
			
Intercept and variables	Estimate	*z*-value	OR (95%)	*P*-value
Intercept	−9.713	−2.981	0.00 (0.00, 0.02)	0.003*
Ln-calprotectin	0.086	2.222	3.38 (1.37, 9.47)	0.026*
PsA disease duration (years)	1.218	2.495	1.09 (1.01, 1.18)	0.013*

*P value < 0.05.

### Validation of the Predictive Model

**FIGURE 3:**
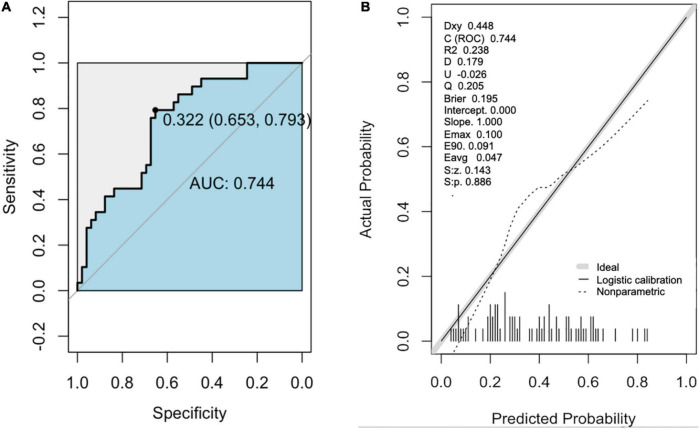
The receiver operator characteristic (ROC) curve and Calibration curve of the nomogram for predicting in patients with PsA. **(A)** ROC curve of the predictive model for CP+. **(B)** Calibration curves of the predictive model for CP+. The black line represents a great fit between the nomogram predicted probability (*x*-axis) and the observed probability of actual diagnosed cases of CP+ (*y*-axis). The dotted line represents performance of the present nomogram. Closer fit between the two lines indicates a higher prediction accuracy. CP+, the presence of carotid plaque.

The area under the receiver operating characteristic (AUC) for the predictive model was 0.744 (95% CI [0.59, 0.80]; *P* = 0.037) with 69% sensitivity and 79% specificity (Figure 3A and Table 3). In addition, the accuracy, PPV, and NPV were 75.6, 84, and 57%, respectively (Table 3). A calibration curve showed a good degree of fit and another calibrated marker, Hosmer-Lemeshow test was also optimal (*P* = 0.061) (Figure 3B and Table 3).

**TABLE 3:** Discrimination and calibration assessment of CP+ nomogram model selected by multivariate and LASSO regression.

	Value (95% CI)	*P*-value
**Goodness of fit**	
AIC	94.0	–
**Discrimination of nomogram predictive model**	
AUC	0.744 (0.59, 0.80)	0.037*
Sensitivity (%)	65.3	–
Specificity (%)	79.3	–
Accuracy (%)	75.6	–
PPV (%)	84.2	–
NPV (%)	57.5	–
**Calibration of nomogram predictive model**	
HL		0.061

*P value < 0.05. CP+, carotid plaque; AUC, area under the curve; PPV, positive predicted value; NPV, negative predicted value; HL, Hosmer-Lemeshow chi-square statistical test.

## Discussion

In order to determine whether serum calprotectin was a useful biomarker of PsA patients with increased CV risk, we used the presence of CP as a surrogate marker. This is the first report on the association between the presence of CP and serum calprotectin level in patients with PsA. Moreover, subclinical carotid atherosclerosis was associated with the inflammatory burden (as reflected by the disease duration) in these patients.

The association between PsA and increased CV risk is well established, however, only limited data investigated the underlying mechanisms. In PsA patients, serum calprotectin level was independently associated with the presence of CP, probably involving platelet and endothelial cells activation. Calprotectin levels correlated with platelet aggregation and activation as well as serum thromboxane B2 levels in stable coronary artery disease patients (24). Furthermore, calprotectin can bind to endothelial cells through carboxylated glycans and Toll-like receptor 4 (TLR4) leading to cell activation (25, 26), an increase in permeability of endothelium with leukocyte extravasation (27), and triggering of endothelial cell apoptosis (28), resulting in vascular and tissue damage. All these factors may subsequently lead to plaque formation.

Least absolute shrinkage and selection operator regression analysis was performed to actively select from a large and potentially multicollinear set of variables in the regression, resulting in two variables which were put into the multivariate logistic regression model. The Ln-calprotectin and PsA disease duration were independent explanatory variables associated with the presence of CP. The normogram developed for discriminating the presence of CP was a easy reference for clinical practice, with good accuracy, sensitivity and specificity (AUC 0.744, sensitivity 65%, specificity 79%).

Consistent with our results, a recent study reported that the PsA disease duration was an independent predictor to the total plaque volume (TPV) and segment involvement score evaluated by coronary computed tomography angiography (CCTA). Nonetheless, they did not find any relationship between TPV and metabolic syndrome (including a history of obesity, hypertension, dyslipidemia, and insulin resistance) (29). Our study results suggested that serum calprotectin level was able to discriminate PsA patients with and without CP while traditional CV risk scores were not selected by the regression analysis. Indeed, these CV risk scores only had moderate discriminating abilities to identify inflammatory arthritis patients with elevated CV risk (9, 30, 31). Proinflammatory cytokines play a major role in plaque remodeling and fibrous cap thinning leading to CV events. Yet, most CV risk scores do not take inflammation into consideration.

C-reactive protein and erythrocyte sedimentation rate levels are widely used as markers of acute inflammation in PsA. Nevertheless, these acute phase reactant levels are usually in the normal range despite patients having high disease activity (32). Serum calprotectin level has been reported as a more sensitive biomarker of joint disease and polymorphic disease manifestations in PsA than CRP (33). In the current study, serum calprotectin level correlated with the presence of CP as well as the inflammatory burden (as reflected by the disease duration). In contrast, CRP and ESR levels were not associated with the presence of CP and disease duration (data were not shown), suggesting that serum calprotectin was a better biomarker in reflecting the inflammatory and atherosclerotic burden in PsA than a one-off measurement of these inflammatory markers. The serum calprotectin levels are influenced by local inflammation and synovial fluid calprotectin levels (34). Thus, it was hypothesized that serum calprotectin levels reflect the amount of synovial inflammation. In early PsA, serum calprotectin correlates with ultrasound measures of disease activity (35). This observation is further supported by a correlation between calprotectin and the ultrasound power doppler score, with calprotectin outperforming CRP (21). These observations might suggest that serum calprotectin could also be a biomarker of disease severity and prognosis in PsA, but this has to be further evaluated in a longitudinal study. Biological treatment induced a drastic reduction of calprotectin expression in skin biopsies from treated psoriasis patients, correlating with PASI reduction, suggesting that calprotectin may play a crucial role as a significant marker of inflammation in psoriatic disease. Whether its reduction of expression may be considered a favorable prognostic marker in PsA would need to be confirmed in future prospective studies.

There are some potential limitations in this cross-sectional study. First, future prospective studies will be needed to assess the association between changes in serum calprotectin level and progression of CP in PsA patients. Second, studies using 3D-US should be considered in the future to accurately assess the correlation between CP volume and calprotectin levels. In addition, the association between serum calprotectin level and TPA or plaque vulnerability would need to be addressed further. Third, majority of our patients had low disease activity (only six patients had moderate or high disease activity and the median value of DASPA was 2.5). Our results may not be applicable to patients with moderate or high disease activity. Fourth, a validation cohort would be required to evaluate the performance of the prediction model. In view of the limited patient number, only the internal validation by the k-fold cross-validation was performed. Last but not least, whether elevated calprotectin should be considered a poor prognostic marker requiring early treatment using bDMARDs in PsA patients in order to prevent long-term damage accrual and CV event would deserve further studies.

## Conclusion

Serum calprotectin level may be a useful biomarker associated with a high inflammatory burden and the presence of subclinical carotid atherosclerosis in patients with PsA.

## Data Availability Statement

The raw data supporting the conclusions of this article will be made available by the authors, without undue reservation.

## Ethics Statement

The studies involving human participants were reviewed and approved by The Joint Chinese University of Hong Kong – New Territories East Cluster Clinical Research Ethics Committee written informed consents were obtained from all patients according to the Declaration of Helsinki. The ethical approval number is 2017.683. The patients/participants provided their written informed consent to participate in this study.

## Author Contributions

L-ST and IC: study design. L-ST, EL, ML, AL, BY, PW, and HS: data collection. IC, HM, and JL: data analysis. HM, IC, HS, and L-ST: drafting of manuscript. All authors were involved in drafting the article or revising it for important intellectual content, and approved the final version to be submitted for publication.

## Conflict of Interest

The authors declare that the research was conducted in the absence of any commercial or financial relationships that could be construed as a potential conflict of interest.

## Publisher’s Note

All claims expressed in this article are solely those of the authors and do not necessarily represent those of their affiliated organizations, or those of the publisher, the editors and the reviewers. Any product that may be evaluated in this article, or claim that may be made by its manufacturer, is not guaranteed or endorsed by the publisher.

## Footnotes

1
https://www.rstudio.com/
