# Supplementary material for: Serum Calprotectin Level Is Independently Associated With Carotid Plaque Presence in Patients With Psoriatic Arthritis
Source: Front Med (Lausanne). 2022 Jul 8;9:932696. doi: 10.3389/fmed.2022.932696 (PMC9305068; doi:10.3389/fmed.2022.932696)
Supplement: Supplementary file 1 [file Data_Sheet_1.docx]

Supplementary Material

# Supplementary Figures and Tables

## Supplementary Figures


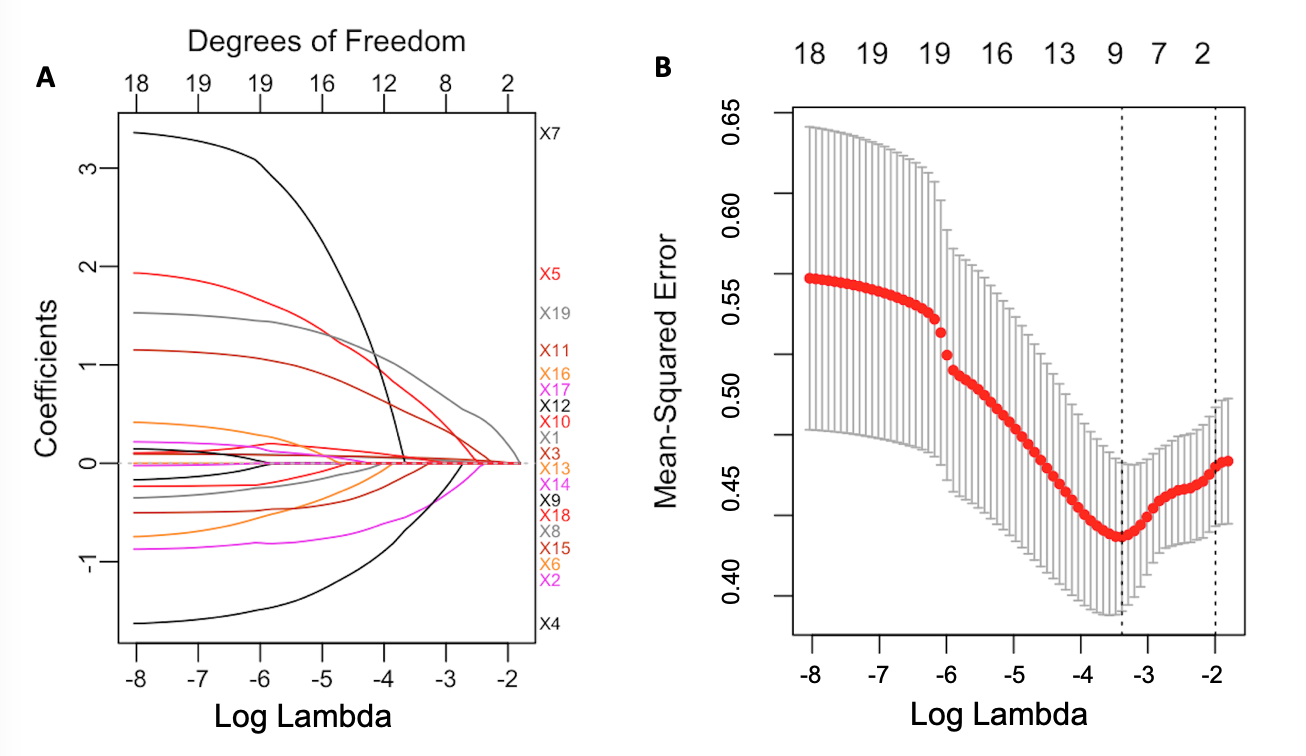


**Supplementary Figure 1.** Supplementary Figure 1. LASSO-Logistic regression choosing the variables for predicting the presence of carotid plaque. **(A). LASSO coefficient profiles of the 19 variables.** A vertical line was drawn as the coefficients of each predictor when the 19 predictors were included in the LASSO regression model.

**(B). The cross-validation results.** The 10-fold cross-validation was used to choose the best lambda. The value in the middle of the two dotted lines is the range of the positive and negative standard deviations of log(λ). The dotted line on the left indicated the log(λ) value when the mean cross-validated error of the model is minimized. The right dotted line is the value of log(λ) that gives the most parsimonious model when the cross-validated error is within one standard error of the minimum. Two variables were selected when log(λ) = -1.99.

**1.2 Supplementary Tables**

| Supplementary Table 1. Correlation between inflammatory burden and subclinical atherosclerosis. | | | | | | | | | | | | |
| --- | --- | --- | --- | --- | --- | --- | --- | --- | --- | --- | --- | --- |
|  |  | Age | Gender | PsA disease duration | CRP | ESR | FRS | Ln-calprotectin | DASPA | TJ count | SJ  count | mean cIMT |
| Age | rho | 1.000 | .050 | .284^*^ | .103 | .292^**^ | .649^**^ | .129 | .165 | .214 | .148 | .427^**^ |
|  | *P value* | . | .666 | .012 | .370 | .010 | .000 | .261 | .152 | .060 | .195 | .000 |
| Male gender | rho | .050 | 1.000 | .044 | -.047 | .417^**^ | -.433^**^ | -.109 | .196 | .261^*^ | -.051 | -.133 |
|  | *P value* | .666 | . | .703 | .686 | .000 | .000 | .340 | .087 | .021 | .660 | .272 |
| PsA disease duration | rho | .284^*^ | .044 | 1.000 | .168 | .157 | .185 | .280^*^ | -.022 | -.041 | -.005 | .046 |
|  | *P value* | .012 | .703 | . | .145 | .173 | .106 | .013 | .849 | .724 | .964 | .703 |
| CRP | rho | .103 | -.047 | .168 | 1.000 | .476^**^ | .079 | .176 | .325^**^ | .140 | .212 | -.017 |
|  | *P value* | .370 | .686 | .145 | . | .000 | .495 | .127 | .004 | .225 | .065 | .888 |
| ESR | rho | .292^**^ | .417^**^ | .157 | .476^**^ | 1.000 | .097 | .044 | .386^**^ | .337^**^ | .355^**^ | .224 |
|  | *P value* | .010 | .000 | .173 | .000 | . | .400 | .704 | .001 | .003 | .002 | .064 |
| FRS | rho | .649^**^ | -.433^**^ | .185 | .079 | .097 | 1.000 | .235^*^ | -.003 | .043 | .089 | .593^**^ |
|  | *P value* | .000 | .000 | .106 | .495 | .400 | . | .038 | .977 | .706 | .437 | .000 |
| Ln-calprotectin | rho | .129 | -.109 | .280^*^ | .176 | .044 | .235^*^ | 1.000 | .135 | .065 | .103 | .249^*a^ |
|  | *P value* | .261 | .340 | .013 | .127 | .704 | .038 | . | .243 | .574 | .371 | .038 |
| DASPA | rho | .165 | .196 | -.022 | .325^**^ | .386^**^ | -.003 | .135 | 1.000 | .881^**^ | .720^**^ | .101 |
|  | *P value* | .152 | .087 | .849 | .004 | .001 | .977 | .243 | . | .000 | .000 | .407 |
| TJ count | rho | .214 | .261^*^ | -.041 | .140 | .337^**^ | .043 | .065 | .881^**^ | 1.000 | .554^**^ | .098 |
|  | *P value* | .060 | .021 | .724 | .225 | .003 | .706 | .574 | .000 | . | .000 | .422 |
| SJ count | rho | .148 | -.051 | -.005 | .212 | .355^**^ | .089 | .103 | .720^**^ | .554^**^ | 1.000 | .146 |
|  | *P value* | .195 | .660 | .964 | .065 | .002 | .437 | .371 | .000 | .000 | . | .228 |
| mean cIMT | rho | .427^**^ | -.133 | .046 | -.017 | .224 | .593^**^ | .249^*a^ | .101 | .098 | .146 | 1.000 |
|  | *P value* | .000 | .272 | .703 | .888 | .064 | .000 | .038 | .407 | .422 | .228 | . |
| ** *P* value <0.01; **P* value <0.05.  ^a^ Pearson’s correlation coefficient was used to measure the association between Ln-calprotectin and mean cIMT. The association of other variables was measured using Spearman’s correlation coefficient.  CRP, C-reactive protein; ESR: erythrocyte sedimentation rate; FRS, Framingham risk score; DAPSA, Disease activity in psoriatic arthritis; TJ, Tender joint; SJ, Swollen joint; cIMT, Carotid intima-media thickness. | | | | | | | | | | | | |

| Supplementary Table 2. Variable assignments. | | |
| --- | --- | --- |
| **Variables** | **Risk Factors** | **Assignment** |
| X1 | Age | Continuous variable |
| X2 | Gender | Female = 0, Male = 1 |
| X3 | Disease duration | Continuous variable |
| X4 | History of HT (including anti-HT drug use) | Yes =1. No = 0 |
| X5 | History of hyperlipidemia (including statin use) | Yes =1. No = 0 |
| X6 | History of DM (including hyperglycemic drug use) | Yes =1. No = 0 |
| X7 | Waist to hip ratio | Continuous variable |
| X8 | Current smoker | Yes =1. No = 0 |
| X9 | Tender joint count | 0-68 |
| X10 | Swollen joint count | 0-66 |
| X11 | Dactylitis number | 0-20 |
| X12 | DAPSA | Continuous variable |
| X13 | CRP | Continuous variable |
| X14 | ESR | Continuous variable |
| X15 | DMARDs use | Yes =1. No = 0 |
| X16 | NSAIDs use | Yes =1. No = 0 |
| X17 | Biologic use | Yes =1. No = 0 |
| X18 | Corticosteroid use | Yes =1. No = 0 |
| X19 | Ln-calprotectin | Continuous variable |
| HT: Hypertension; DM: Diabetes mellitus DAPSA: disease activity in psoriatic arthritis; CRP: C-reactive protein; ESR: erythrocyte sedimentation rate; csDMARDs: conventional synthetic disease-modifying antirheumatic drugs. bDMARDs: biologics disease-modifying antirheumatic drugs; NSAID: nonsteroidal anti-inflammatory drugs. | | |
